# Decoding voluntary movements and postural tremor based on thalamic LFPs as a basis for closed-loop stimulation for essential tremor

**DOI:** 10.1016/j.brs.2019.02.011

**Published:** 2019

**Authors:** Huiling Tan, Jean Debarros, Shenghong He, Alek Pogosyan, Tipu Z. Aziz, Yongzhi Huang, Shouyan Wang, Lars Timmermann, Veerle Visser-Vandewalle, David J. Pedrosa, Alexander L. Green, Peter Brown

**Affiliations:** a Medical Research Council Brain Network Dynamics Unit at the University of Oxford, OX1 3TH, Oxford, United Kingdom; b Nuffield Department of Clinical Neurosciences, John Radcliffe Hospital, University of Oxford, OX3 9DU, Oxford, United Kingdom; c Nuffield Department of Surgical Sciences, University of Oxford, Oxford, United Kingdom; d Neural and Intelligence Engineering Center, Institute of Science and Technology for Brain-Inspired Intelligence, Fudan University, Shanghai, China; e Department of Neurology, University Hospital of Gießen and Marburg, Marburg, Germany; f Department of Stereotactic and Functional Neurosurgery, University Hospital Cologne, 50924, Cologne, Germany

**Keywords:** Local field potential, Essential tremor, Ventral intermediate thalamus, Deep brain stimulation

## Abstract

**Background:**

High frequency Deep brain stimulation (DBS) targeting motor thalamus is an effective therapy for essential tremor (ET). However, conventional continuous stimulation may deliver unnecessary current to the brain since tremor mainly affects voluntary movements and sustained postures in ET.

**Objective:**

We aim to decode both voluntary movements and the presence of postural tremor from the Local field potentials (LFPs) recorded from the electrode implanted in motor thalamus for stimulation, in order to close the loop for DBS so that stimulation could be delivered on demand, without the need for peripheral sensors or additional invasive electrodes.

**Methods:**

LFPs from the motor thalamus, surface electromyographic (EMG) signals and/or behavioural measurements were simultaneously recorded in seven ET patients during temporary lead externalisation 3–5 days after the first surgery for DBS when they performed different voluntary upper limb movements. Nine different patients were recorded during the surgery, when they were asked to lift their arms to trigger postural tremor. A machine learning based binary classifier was used to detect voluntary movements and postural tremor based on features extracted from thalamic LFPs.

**Results:**

Cross-validation demonstrated that both voluntary movements and postural tremor can be decoded with an average sensitivity of 0.8 and false detection rate of 0.2. Oscillatory activities in the beta frequency bands (13–23 Hz) and the theta frequency bands (4–7 Hz) contributed most to the decoding of movements and postural tremor, respectively, though incorporating features in different frequency bands using a machine learning approach increased the accuracy of decoding.

## Highlights

• Essential tremor mainly affects voluntary movements and sustained postures.
• Both voluntary movements and postural tremor can be decoded using LFPs recorded from electrodes implanted in motor thalamus for stimulation.
• This information can potentially be used to close the loop for DBS for the treatment of essential tremor.
• Closed-loop DBS for ET can be achieved without the need for peripheral sensors or additional invasive electrodes.

## Introduction

Continuous high frequency Deep brain stimulation (DBS) targeting ventral-intermediate thalamus is an effective therapy for medically refractory essential tremor (ET) [[1], [2], [3], [4]]. However, as many as 70% of patients lose the benefit of DBS over time [5], due to disease progression or habituation to stimulation [6]. These circumstances often require an increase in the energy delivered through increased amplitude, frequency, and/or increased pulse width, which is commonly associated with more pronounced adverse effects, resulting in slurred speech, unpleasant sensations, incoordination and walking difficulty [7]. Furthermore, tremor in ET is typically intermittent, predominantly occurring during voluntary movements and sustained postures [[8], [9], [10]], suggesting that stimulation could be more focussed in time to control symptoms.

Closed-loop stimulation, in which stimulation parameters are automatically adjusted to stimulate on demand, is seen as a potential breakthrough for the treatment of essential tremor [2,11]. Prior studies of closed-loop DBS for ET have used wearable inertial sensors [12,13] and/or surface electromyography (EMG) [14] to provide feedback for the control of the stimulator. While wearable sensors provide reliable measurements of tremor, the wireless communication between the neurostimulator and the external sensors introduces a potential vulnerability to the system due to breaks in transmission. In more recent studies [15,16], movement-related signals have been recorded using a strip of intracranial electrodes implanted over the surface of the motor cortex. These signals are used to detect movements and to activate the DBS [8]. A recent single case study has shown that this approach can reduce tremor during writing and spiral drawing [16]. Nevertheless, the use of cortical strip electrodes introduces further instrumentation and additional cost. It is therefore still not standard clinical practice to implant cortical strip electrodes in patients undergoing therapeutic DBS for the treatment of tremor. In addition, triggering DBS at the detection of movements may not provide sufficient control of tremor during sustained posture which is also an important aspect of ET [9].

Local field potentials (LFPs) recorded in the motor thalamus may contain information related to both voluntary movements and postural tremor. Movement-related potentials in ventral intermediate (ViM) thalamic LFPs were observed with a similar latency as in the cortex [17,18]. In the frequency domain, reduction in the power of beta oscillations (14–30 Hz) and increase in a broad gamma frequency range (55–80 Hz) were reported in ViM thalamus during movements [17,19]. Ventral thalamic nucleus also expresses activities relating to ongoing tremor. For example, populations of neurons in the ViM thalamus exhibit tremor-frequency activity during tremor but not during rest [20]. Increased synchronisation at tremor and double tremor frequency in the ventral lateral posterior (VLP) nucleus of the thalamus has also been associated with the presence of tremor [[21], [22], [23]].

Here we show that both voluntary movements and postural tremor can be decoded based on LFPs recorded from the same electrodes that are implanted in motor thalamus for therapeutic DBS. Importantly for the practical application of the proposed methods in decoding movements and triggering DBS, the classifier identified based on data recorded while patients performed pre-defined cued movements can also be used to decode different natural movements such as drawing and pointing. Additionally, we show that postural tremor can also be decoded but the features for decoding postural tremor are different from those for decoding movements, suggesting a separate model would be required in order to deliver stimulation during postures that provoke tremor without further voluntary movements. Together these results pave the way for adaptive DBS based on LFP signals recorded from motor thalamus, without additional intracranial electrodes or external sensors.

## Materials and methods

### Participants

LFPs were recorded from seven ET patients (26–74 years old, 4 females) after obtaining informed written consent to take part in the study, which was approved by the local ethics committee. These participants underwent surgery for the implantation of DBS electrodes targeting the ViM thalamus at the Department of Functional Neurosurgery at the John Radcliffe hospital, Oxford. Leads were temporarily externalised following electrode implantation and recordings were performed 3–5 days later, before final implantation and connection to the implantable pulse generator. Three patients received unilateral implantation whereas the other four patients received bilateral implantation, affording recordings from 11 ViM thalami in total. Details of the patients are reported in Table 1.

**Table 1:** **Patient details and motor tasks that have been tested.** Patients in Oxford (Ox**) were recorded post-operatively during temporary lead externalisation and different motor tasks that have been tested. Patients in Cologne (Cl**) were recorded inside the theatre during the surgery.

ID	Age	Gender	Recorded hemisphere	Recording electrode	Pre-defined movements	Cross Task Validation
Ox 1	35	M	Left ViM	3389; Medtronic	Cued Gripping Force (right hand only)	
Ox 2	54	F	Bilateral ViM	3387; Medtronic	Cued Joystick Movement (both hands separately)	
Ox 3	62	F	Left ViM	3387; Medtronic	Cued button pressing (right hand only)	Drawing (right hand only)
Ox 4	26	M	Bilateral ViM	3387; Medtronic	Self-paced continuous finger tapping (both hands)	Drawing (both hands separately)
Ox 5	37	F	Left ViM	3387; Medtronic	Self-paced continuous flexion/extension of right wrist	Drawing (right hand only)
Ox 6	65	F	Bilateral ViM	6180; St. Jude Directional	Cued Gripping Force (both hands separately)	Self-paced reach and grasp + Self-paced pegboard movement (right hand only)
Ox 7	74	M	Bilateral ViM	3389; Medtronic	Cued Gripping Force + Self- paced continuous tapping (both hands separately)	Drawing + Self-paced pegboard movement (right hand only)
Cl 1	64	M	Right VLp	micro-macroelectrode (LFPs recorded from the macro contacts were used for analysis)	Keep arm rest for 30–60 s and then elevate and hold their forearm at an angle of ∼30° and to spread their fingers for 30–60 s	
Cl 2	52	M	Right VLp			
Cl 3	75	F	Left VLp			
Cl 4	71	F	Left VLp			
Cl 5	73	F	Bilateral VLp			
Cl 6	67	F	Bilateral VLp			
Cl 7	69	F	Bilateral VLp			
Cl 8	62	M	Left VLp			
Cl 9	72	M	Bilateral VLp			

Both postural tremor and action tremor pose challenges in everyday life activities for patients affected by essential tremor. Here we explore the potential of the ViM thalamus LFP to provide a feedback signal capable of controlling DBS so that it focusses on periods when tremor is likely or present. It is therefore important that both voluntary movements likely to trigger tremor, and postural tremor itself, can be decoded from the signal, to ensure that the DBS is switched on in both situations. Tremor may be considerably improved over the days following electrode implantation due to the stun effect of surgery [24,25], and prominent postural tremor was only observed in one of the seven patients recorded post-operatively in Oxford. This case (Ox7) still displayed significant postural tremor with amplitude larger than 2 cm when holding the arms abducted, up in the air with elbows flexed and the fingers of both hands pointing towards each other. To test whether we can decode the presence of postural tremor from thalamic LFPs, intra-operative data from another cohort of ET patients who underwent bilateral implantation of DBS electrodes into the thalamus at the Department of Stereotactic and Functional Neurosurgery in Cologne were also analysed. This afforded an opportunity to record from the micro-macroelectrodes used during target identification before the definitive DBS electrode implantation. The micro-macroelectrodes (INOMED MER System 2.4 beta) consist of a microelectrode tip (diameter 4 μm) and a macroelectrode ring (diameter 800 μm) 1 mm above the tip. The electrodes have narrower diameter than the definitive DBS electrodes, and are less prone to induce a stun effect [24]. The macroelectrodes are low impedance electrodes (at about 1 kΩ) and were used to record the LFPs once the target location was reached. Raw data were first visually inspected, and those with severe artefact were excluded. The average standard deviation of the recorded LFP signals was around 8 μA across all patients. Low frequency variations or broadband activities with amplitude larger than 30 μA were treated as artefact. Only data sets with at least 30 s of artefact-free recording from each condition (rest and postural tremor) were included for final analysis in this study. These were from 12 ViM thalamus recordings from 9 patients (5 females, 67.4 ± 2.4 years old). The study was approved by the local ethics committee in Cologne and carried out in accordance with the Declaration of Helsinki. Detailed information about the patients and different aspects of the data have been previously described [22,26].

The exact procedures of the surgery in the two centres are described in Supplementary methods.

### Experimental design and recording

During the post-operative recordings in Oxford, patients were seated in a chair in front of a desktop monitor and performed different upper limb movements. In order to test the versatility of the proposed methods in detecting different movements, several motor tasks with different durations and different muscle effectors, such hand gripping, finger tapping, finger joystick movements were used across different patients (see Table 1, supplementary methods and Supplementary Fig. 1 for more details of the motor tasks). In order to further test the within-subject generalisability of the classifier for detecting movements, five of the seven patients performed some other self-paced movements such as spiral drawing, reaching and grasping (Table 1). Importantly, these movements were different from those used to train the classifier, so as to see if the classifier trained on pre-defined movements can decode other self-paced movements the patient might perform in everyday life.

Patients in Cologne were asked to perform a simple motor paradigm inside the operation theatre. This consisted of two conditions: (1) supine patients rested their arm in a comfortable position for 30–60 s; (2) supine patients were asked to elevate and hold their forearm contralateral to the implantation side at an angle of ∼30° and to spread their fingers for 30–60 s. Subjects performed the tasks sequentially while awake after at least 15 min of withdrawal of sedation (remifentanil and/or propofol). Patients performed the task without speaking or performing any other activities.

ViM thalamic local field potentials, electromyography (EMG) and behavioural measurements such as gripping force, joystick positions and accelerometer attached to hand were simultaneously recorded (details presented in Supplementary Methods).

### LFP pre-processing and feature extraction

The monopolar LFP data were re-referenced offline to obtain more spatially focal bipolar signals by subtracting the data from neighbouring electrode contacts [27]. The data were band-pass filtered between 1 Hz and 200 Hz (Butterworth filter, filter order = 4) and down-sampled to 1000 Hz. Time-frequency decomposition was obtained on each down-sampled bipolar channel by applying continuous Morlet wavelet transforms with a linear frequency scale ranging from 1 Hz to 195 Hz and constant number (= 6) of cycles across all calculated frequencies. Relative power was then calculated for each frequency by normalizing the absolute power by its average across time for each channel: (power – average power)/average power * 100. Average movement-related modulation in the power spectra was calculated for each bipolar channel by taking the average of each 2 s epoch aligned to movement onset. The bipolar channel in each electrode with the highest modulation in the 15–35 Hz within the [-1 s, 1 s] window aligned to the movement onset (max-min) was selected for further processing. This was motivated by evidence linking maximal beta band activity and re-activity to the dorsal (motor) region of the STN [[28], [29], [30], [31], [32]]. For postural tremor detection, where LFP measurements were recorded from multiple micro-macroelectrodes, the decoding was tested based on each LFP measurement. The channel with best decoding accuracy (the largest AUC value) was selected to report for that side.

A logistic regression (LR) model (more details in Supplementary Methods) was used to predict the probability of the presence of movements or tremor at the current time point t (p(t)) based on a linear combination of features extracted from pre-processed LFPs. Informed by our previous work, the power of oscillatory activities in different frequency bands over a short time window can be potential predictive features for decoding movements [[33], [34], [35]]. Here, the average power of eight non-overlapping frequency bands were quantified after wavelet transformation applied to the selected thalamic LFPs contralateral to the moving hand: 1–3 Hz, 4–7 Hz, 8–12 Hz, 13–22 Hz, 23–34 Hz, 35–45 Hz, 56–95 Hz and 105–195 Hz. The mean power in each of these bands was calculated over a moving time window with window length of 250 ms and overlap ratio of 60%, and then normalized against the mean power of that frequency band over the recording session. Predictive features over 10 consecutive moving windows (equivalent to 1 s preceding the current time point) were included as predictor variables. This time window was selected since movement-related potentials in ViM thalamic LFPs can be observed up to one second before the actual movement [17,18]. In addition, only data preceding the decision-making time point was used for decoding to ensure that the algorithms proposed here can be implemented in real-time. This resulted in 80 predictor variables (8 frequency bands * 10 moving windows) as the inputs for the logistic regression model. The output of the LR classifier was updated every 100 ms.

### Classifier training, evaluation and cross-task validation

Five-fold cross validation was performed for each recording session (more details in Supplementary Methods and Supplementary Fig. 2). This was used to evaluate the capacity of the classifier to decode the same pre-defined movement recorded within the same recording session. In order to further evaluate the across-session and across-task generalisability of the LR based classifier, the classifier trained with data recorded during pre-defined movements was tested for decoding other types of self-paced movements in five patients. The decoded movement probability reported hereafter are ‘test’ results, with the model trained on one dataset and applied on different data. Labelling of movement states based on behavioural measurement (detailed in Supplementary Methods) was used as the ‘ground truth’ in the training and testing.

To evaluate the performance of the classifiers, the ROC was plotted, the area under the curve (AUC) and the sensitivity (percentage of movement time that was accurately detected) were quantified and presented. In addition, the detection rate and the detection latency of individual movements was also quantified. To do so, the LR classifier output around a time window between −2.5 s and +2.5 s around each individual movement onset was evaluated. A movement was treated as detected if within this time window, the LR output started from a value lower than the threshold of 0.4, increased to values higher than the threshold and stayed above this threshold for at least 500 ms. The percentage of successfully detected movements in all movements recorded in a task session was quantified as *detection rate*. The time of the LR output first exceeded the threshold relative to the actual movement onset was quantified as the *latency* of the detection.

### Contribution of different LFP features in movement decoding

The percentage of contribution of LFP features in different frequency bands and different time lags (%C(k,m)) and the percentage of contribution from features in each frequency band $\left(\%C_{freq}\left(k\right)\right)$ for movement or tremor decoding are calculated based on the absolute value of the weight attributed to each predictive feature $\left(w_{k,m}\right)$:

$$\%C\left(k,m\right)=\frac{\left|w_{k,m}\right|}{\sum_{m=0}^{M}\sum_{k=1}^{K}|w_{k,m}|}$$

$$\%C_{freq}\left(k\right)=\frac{\sum_{m=0}^{M}|w_{k,m}|}{\sum_{m=0}^{M}\sum_{k=1}^{K}|w_{k,m}|}$$

The importance of frequency bands for decoding movements was also evaluated by comparing the AUC values as the performance of the classifier after removing features of specific frequency bands.

## Results

### Activities in ViM thalamic LFPs are modulated by movements

**Fig. 1:**
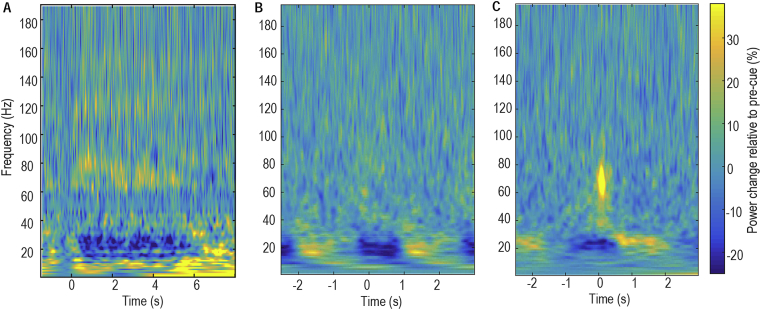
**Examples of power changes induced by movements in ViM thalamus contralateral to the voluntarily moved hand.** Changes were quantified relative to the average of the whole recording session and averaged across all trials during A) hand gripping for patient Ox1; B) finger joystick movements for patient Ox2; C) button pressing for patient Ox3. Time zero represents onset of individual movements.

Average time-evolving power spectra of changes in ViM thalamic LFPs induced by movements were derived by aligning the normalized power spectra to all contralateral movement onsets and averaging across all individual movements in an experimental run (Fig. 1). This identified power increase in the theta/alpha band (4–12 Hz), power reduction in the beta range (13–34 Hz), and power increases in the mid gamma (56–95 Hz) and high-gamma/high-frequency (105–195 Hz) ranges during movements. However, the peak frequencies and ranges of movement-related changes varied from patient to patient.

### Both cued brief movements and blocks of continuous movements are detected

**Fig. 2:**
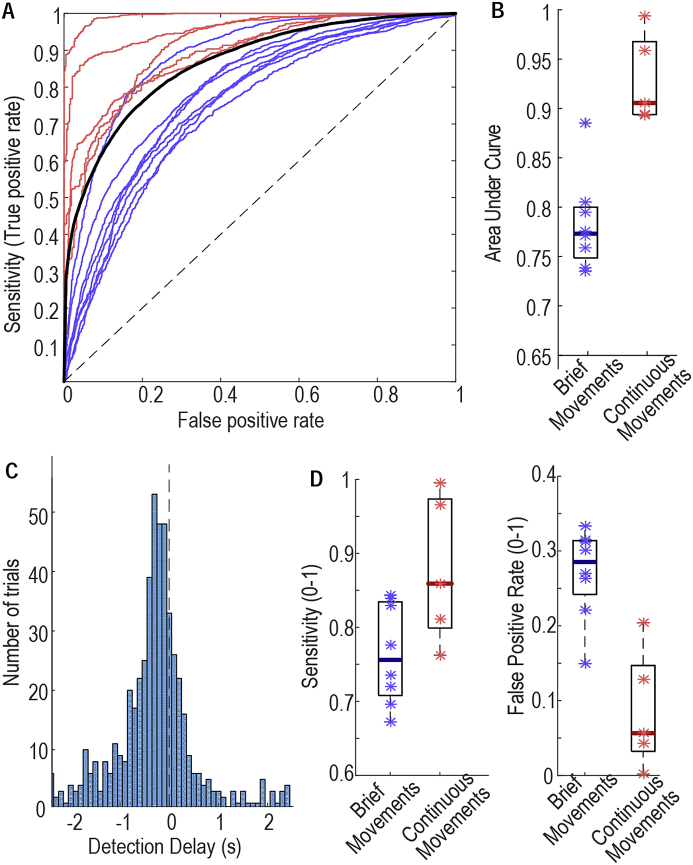
**Evaluation of movement detection.** A) ROC plots showing sensitivity against the false positive rate for the different possible thresholds used for decoding. Blue and red thin lines show the ROC curves of individual cases for cued brief movements and self-paced continuous movements, respectively. The thick blue line shows the ROC averaged across all cases. B) Area Under the Curve (AUC). C) Histogram of detection delays of individual movements. Zero is movement onset. D) Sensitivity and false positive rate for detecting brief and continuous movements, respectively, with a constant decision threshold of 0.4. (For interpretation of the references to colour in this figure legend, the reader is referred to the Web version of this article.)

The within-task cross-validation tests showed that ViM LFPs could be used to detect hand gripping despite the variation in force generated in each grip (Supplementary Fig. 3A), as well as joystick movements (Supplementary Fig. 3B) or button pressing (Supplementary Fig. 3C), despite the short duration of individual movements. The same approach could also detect blocks of self-paced continuous movements (Supplementary Fig. 4). In all cases, the LR-based classifier output increased when movements began and remained high until the movement stopped. The AUC ranged between 0.74 and 0.89 for cued brief movements, and between 0.89 and 0.99 for blocks of continuous movements (Fig. 2A&B). With a constant threshold of 0.4, 95.6% ± 2% (mean ± SEM across different test session) of individual movements were detected with a mean latency of −300 ms. The negative detection latency meant that the classifier output exceeded the decision threshold 300 ms before the actual movement onset. With the decision threshold of 0.4, the decoding sensitivity was between 0.67 and 0.84 for brief movements and between 0.76 and 0.99 for continuous blocks of movements. The corresponding false positive rate was between 0.15 and 0.33 for brief movements, and between 0.002 and 0.20 for continuous movements. If DBS was actuated when the movement decoder output exceeded 0.4, the DBS would be switched on 80.8% ± 2.6% of the time when the patients were making any voluntary movements, and the DBS would be switched on 20.0% ± 3.0% of the time when the patients were at rest. It seems that decoding performance is better for continuous movements. As shown in Fig. 2C, a large percentage of the brief movements were detected with negative delays, which means they were detected before the actual movements happened. Provided this anticipation is not too great then the earlier detection of movement is beneficial for the clinical implementation of closed-loop control, because DBS can be triggered and develop its effect before any tremor develops. However, that anticipation will be counted as a ‘false positive’ in the present analysis. For brief movements, the percentage of time quantified as ‘false positive’ would be larger than for the continuous movements. This may be the reason why the detection of continuous movement has a higher sensitivity and lower false detection rate.

### Contribution of different ViM LFP features in decoding of movement

**Fig. 3:**
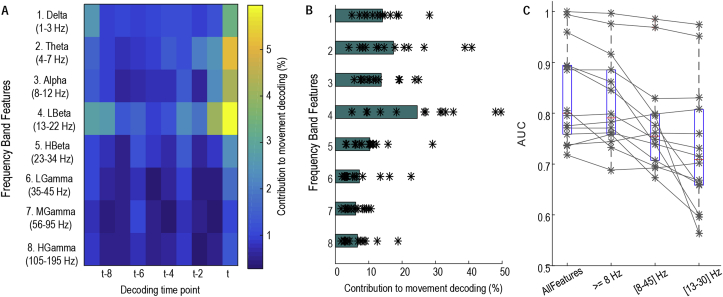
**Features important for movement decoding.** A) Weight (absolute value) distribution across features indicating low beta activities (13–22 Hz) at the most recent time window (t) and the time window just before (t-1) each decision-making time point contributed most to decoding movements. B) Sum of weights for features of different frequency bands, ordered as in A. C) AUC values were not much affected by removing features lower than 8 Hz. AUC values when considering only 8–45 Hz, and only beta band activities (13–34 Hz) are also shown.

Averaged across all test recording sessions, activities in the low beta band (13–22 Hz) contributed most to the movement decoding, and this was followed by activities in the theta (4–7 Hz), delta (1–3 Hz), alpha (8–12 Hz), and high beta band (23–34 Hz) in order of contribution (Fig. 3A&B). The decoding performance remained high after removing activities lower than 8 Hz, which could potentially be contaminated by movement artefacts, from the Logistic regression. Similar decoding performance was reached after further removing activities higher than 45 Hz, which might be contaminated by stimulation artefact if DBS were switched on. However, if only the broad-band beta activity and its history were included, the decoding performance was noticeably lower (Fig. 3C).

### Cross-task validation of movement detection

**Fig. 4:**
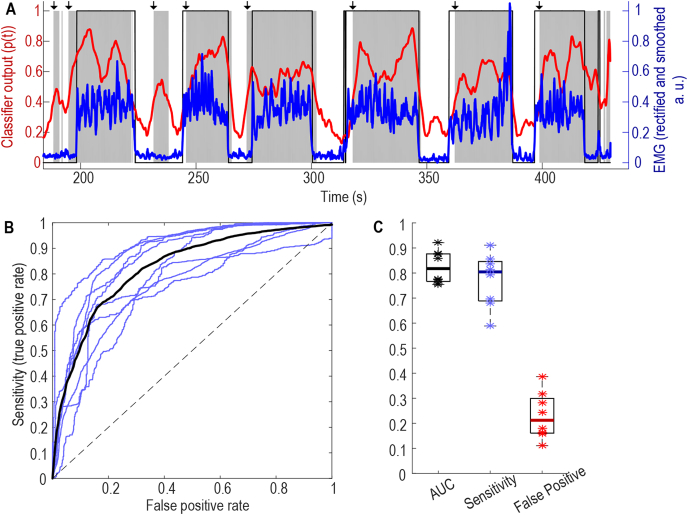
**Results of cross task validation test.** A) Models trained based on data recorded when the patient performed cued hand gripping can detect self-paced pegboard movement in the same patient (Ox6). Blue lines show normalized EMG measurements; thin black lines show time points with movement labelled according to EMG; thick red line shows output from the classifier, and shaded grey areas show the time points with decoded movement probability larger than 0.4. Black arrows at the top indicate the timings when the classifier output crossed the 0.4 threshold and remained higher than 0.4 for more than 200 ms. B) ROC of eight individual cross-task test sessions in blue from 5 patients and average across all test sessions in black; C) AUC values, sensitivity and false positive detection rate at the threshold of 0.4. * indicates values from individual test sessions, thick horizontal lines show the average across all test sessions. (For interpretation of the references to colour in this figure legend, the reader is referred to the Web version of this article.)

The LR-based classifier trained using data recorded while the subjects performed pre-defined cued movements decoded other self-paced voluntary movements such as drawing, reaching and picking up objects with high sensitivity (Fig. 4). In all the 8 cross-task validation test sessions from 5 patients, the AUC of the movement detection was 0.82 ± 0.023. With a constant threshold of 0.4, the sensitivity for movement detection was 0.77 ± 0.038 and the false positive rate was 0.23 ± 0.033. If movement detection were used to actuate DBS, DBS would be switched on 77% ± 3.8% of the time when the patients were engaged in free voluntary movements, and DBS would be switched on 23% ± 3.3% of the time when the patients were at rest.

### Postural tremor detection

**Fig. 5:**
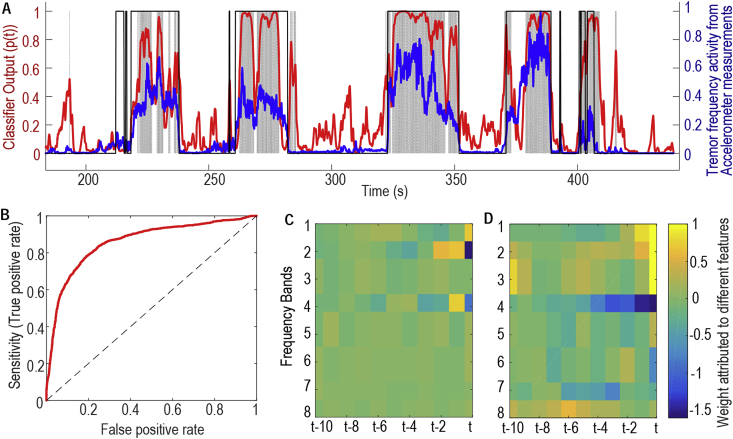
**Postural tremor detection in a representative case (Ox7).** A) Tremor displayed in different postures can also be detected based on ViM LFPs. The blue line shows the normalized tremor frequency power in accelerometer measurements; thin black line shows the time points with tremor judged from accelerometer measurements; the red line shows the classifier output based on ViM LFPs; grey shaded area show the time points with classifier output larger than 0.4. B) ROC plot of the tremor detection shows that 80% detection rate can be achieved with 20% false positive rate. The model optimised for tremor detection (shown in C) is very different from the model optimised for movement detection (shown in D) in the same subject. In C) and D) the x axis is the time window number, where ‘t’ means the most recent time window and ‘t-N’ means the N^th^ time window before the current decision-making time point. Each window has the duration of 250 ms and there is a 150 ms overlap between successive windows. This makes the window ‘t-10’ centre around 1 s before the current time point. Feature labelling and time resolution is the same as in Fig. 3. (For interpretation of the references to colour in this figure legend, the reader is referred to the Web version of this article.)

Tremor was considerably improved in the patients recorded in Oxford in the post-operative period, but one patient recorded post-operatively (Ox7) still displayed significant postural tremor when he was holding the arms abducted, up in the air with elbows flexed and the fingers of both hands pointing towards each other. The postural tremor was evident in the increased 3–7 Hz activity from the accelerometer attached to the hand. However, the model for decoding voluntary movements based on data recorded while the patient performed the cued gripping movements failed to decode postural tremor (AUC = 0.51). A separate model was therefore trained based on data recorded during postural tremor. The within task cross validation showed that the postural tremor could also be decoded based on ViM LFP measurements with the AUC of 0.88 (Fig. 5A&B) if a separate model trained for postural tremor detection was used. With the decision threshold of 0.4, the sensitivity of the detection was 80% and false positive detection was 22%. For the seven blocks of postural tremor recorded, the detection on average anticipated tremor onset by −0.1 ± 0.13 s, ranging from - 0.4 to 0.3 s. However, the LR model for detecting postural tremor, as represented by the weights attributed to different features (Fig. 5C), was very different from that optimised for decoding voluntary movements (Fig. 5D), indicating that separate models might be required to detect voluntary movements and postural tremor in the same subject.

**Fig. 6:**
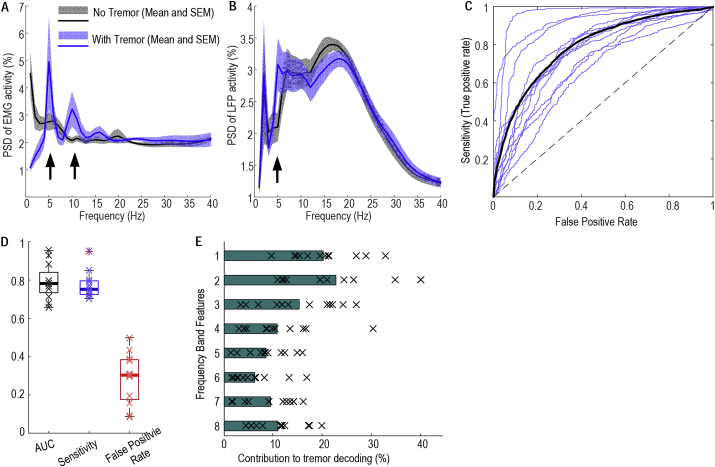
**Postural tremor detection across subjects.** A. Postural tremor was differentiated from rest by increased tremor frequency and double tremor frequency activities (indicated by the arrows) in the EMG; B. Postural tremor was associated with increased tremor frequency activities in the thalamic LFPs (indicated by the arrows). C. ROC curves of tremor detection based on thalamic LFPs (individual sides in thin blue lines and average across all sides in black). D. AUC, Sensitivity and false positive rates. Thick horizontal lines show the mean values and x show data from each individual test session. E. Activities between 4 and 7 Hz contributed most to the tremor decoding, with feature labelling the same as in Fig. 3. (For interpretation of the references to colour in this figure legend, the reader is referred to the Web version of this article.)

In all the patients recorded intraoperatively in Cologne, postural tremor emerged after the elevation of the arm as shown by increased 3–7 Hz activity in the EMG (Fig. 6A). Postural tremor was associated with increased activity in the tremor frequency band (4–7 Hz) in the thalamic LFPs (Fig. 6B). The LR-based classifier based on thalamic LFPs detected postural tremor well above chance-level in all the 12 tested hands from the 9 patients (Fig. 6C). The AUC of tremor detection was 0.79 ± 0.027. With a constant threshold of 0.4, the sensitivity for movement detection was 0.77 ± 0.020 and the false positive rate was 0.29 ± 0.038. The oscillatory activities between 4 and 7 Hz (theta frequency band) in thalamic LFPs contributed most to the tremor decoding, and the AUC of the decoding increased with increasing levels of theta band modulation in thalamic LFPs relative to rest across tested hands (Spearman correlation, r_12_ = 0.825, p = 0.0017).

## Discussion

We have demonstrated that both voluntary movements and postural tremor can be detected based on thalamic LFPs recorded using the same electrode as used for therapeutic stimulation, with an average sensitivity of 0.8 and false positive rate of 0.2. Oscillatory activities in the low beta (13–22 Hz) and theta (4–7 Hz) frequency bands contributed most to detecting voluntary movements and postural tremor, respectively. The movement detection on average preceded movement onset. Critically, the same classifier trained on data recorded during prompted pre-defined movements was also able to detect different self-paced movements, representative of those made during everyday life. However, separate models are required for detecting voluntary movements and postural tremor.

### Implications for closed loop DBS for essential tremor

This study suggest that thalamic LFPs can be sufficient to trigger anticipatory DBS to suppress tremor during action and sustained posture. A previous study monitoring natural hand movements made during everyday life in healthy subjects showed that the hand was essentially at rest for approximately half the time when subjects were awake [36]. Accordingly, actuating DBS only during movement or during postural tremor could lead to up to 50% reduction in the total energy delivered to the brain during awake hours and possibly more once sleep is considered. Compared to previous studies [[12], [13], [14], [15], [16]], our results showed that responsive DBS for essential tremor can be achieved without the requirement of external sensors or additional electrocorticography strips. Using LFP activities recorded from the stimulation electrode for closing the loop for DBS has advantages in minimising the time delays and data loss associated with wireless communication with limb-mounted external sensors, and in minimising the surgical risk of additional invasive instrumentation.

Patients with ET may also develop tremor during sustained postures such as holding an open book. Tremor under these circumstances might not be addressed by triggering DBS with the detection of voluntary movements, whether using thalamic LFPs or electrocorticographic recordings. In addition, decoding failed to detect voluntary movement in a small fraction of active movement trials. So an important aspect of the present study is the ‘failsafe’ procedure of detecting tremor should it develop. In contrast, and to our knowledge, there is still no evidence showing that postural tremor can be detected from cortical signals alone. It should be noted that data reported here for decoding postural tremor were recorded intraoperatively using a micro-macroelectrode system. The macroelectrodes used during intraoperative recordings were different from the definitive DBS electrodes: they have smaller diameters (0.8 mm) compared to DBS electrodes (1.27–1.4 mm) and are therefore likely to cause less of a lesion effect [24,25]. However, the impedances of the two types of electrodes were comparable: the impedance of the macroelectrode tip used during intraoperative recordings was measured at around 1 kΩ; the impedances of DBS electrode contacts have been reported to range from 0.5 to 2 kΩ [37]. Therefore, the activities measured from the two types of electrodes should be roughly similar within the frequency range of interest in the current study (≤195 Hz).

**Fig. 7:**
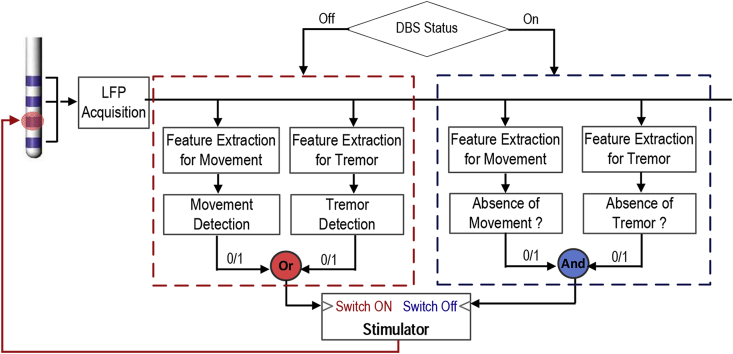
**A proposed schematic for closed-loop DBS for essential tremor based on ViM LFPs.** The detection of movement or tremor is used to actuate the DBS; and the detection of movement offset is used to switch off the DBS, but only provided tremor detection, if present, has ceased.

Nevertheless, there are a few important technical considerations related to using thalamic LFPs for closed-loop DBS. First, all results presented here are based on recordings made with stimulation switched off. Stimulation artefacts lower the signal-to-noise ratio of LFPs recorded when stimulation is on, as shown in Supplementary Fig. 5 for data recorded from a patient diagnosed with tremor dominant Parkinson's disease and receiving DBS targeting ViM thalamus. The detection of movement or tremor onset to start stimulation will not be affected by stimulation artefact. Yet once stimulation is switched on, the classifier needs to detect the offset of movement or tremor to switch off stimulation; here the performance of the classifier may be compromised by the presence of stimulation artefact. Noteworthy, activities in the beta and theta frequency bands recorded from the stimulation electrodes contributed most to movement and tremor detection and can both be monitored even during stimulation, with sufficient filtering and signal processing [[38], [39], [40], [41]]. Supplementary Fig. 5 shows it is possible to decode movements when the high frequency stimulation is switched on with a similar accuracy as with the stimulation was off. It remains to be seen how consistent this is across subjects and whether separate models may be required for detecting movement and tremor offset with simultaneous stimulation. Second, in the approach proposed here, the sensitivity and false-positive rate are dependent on the detection threshold. It is important to consider what is the desired sensitivity and false-positive rate for the best patient outcome in clinical practice. The detection threshold could be further optimised for each patient according to factors such as tolerance to side effects and desirable levels of power saving. Third, we showed that the model optimised for detecting postural tremor was very different from that optimised for detecting voluntary movements. Separate models for detecting movement and postural tremor would be required to ensure that DBS is actuated when either of these two situations is detected for optimal treatment of the disease. Considering all these issues, we propose the framework shown in Fig. 7 to detect both movements and tremor based on ViM LFPs for closed-loop DBS for ET.

### Machine learning based approach vs. single feature threshold-based decision-making

In this study, we used a linear combination of activities in eight non-overlapping frequency bands in the thalamic LFPs for decoding. Even though oscillatory activities in the low beta (13–20 Hz) and theta (4–7 Hz) frequency bands contributed most to the decoding of voluntary movements and postural tremor, respectively, activities in other frequency bands also contributed and increased the decoding accuracy. Thus the present approach may potentially be more effective than threshold-based closed-loop DBS based on activities in one single frequency band, as used in most previous studies [15,16,38], because machine learning based algorithms can automatically attribute appropriate weights to multiple features specific to each patient through optimisation. The algorithm used in this study was based on a multiple linear regression model which can be trained with data recorded over just a few minutes, and can also be easily implemented in real-time for closed-loop DBS applications. It remains to be seen whether other more sophisticated machine learning approaches, which take into account nonlinear relationships, can further improve decoding performance, and whether their implementation is feasible in small, ultra-low power, implantable neurostimulator devices.

### Limitations and caveats

A few general caveats should be borne in mind. First, closed-loop approaches that are based on brain signals assume that these signals do not change significantly over the long life-time of implanted electrodes. So far this has proven to be the case with regard to subthalamic LFPs in patients with Parkinson's disease [42], but this remains to be shown in those with ET. Second, ours is essentially a technical proof-of-principal study. Real-time decoding based on ViM thalamic LFPs, online closed-loop stimulation and the potential advantages of closed-loop DBS based on the detection of movements likely to trigger tremor, and of tremor itself, remain to be tested in acute and chronic clinical trials.

In conclusion, this study demonstrates that LFPs recorded from the ViM thalamus can be used to detect both voluntary movement and postural tremor. This work lays the foundation for future work developing a closed-loop DBS system which continuously updates the decision on whether to stimulate based on activities recorded directly from the point of stimulation, in order to save battery power and minimise side effects in patients with ET.

## Conflicts of interest

The authors have no financial or personal relationship with other people or organisations that could inappropriately influence this work.

## Funding

This work was supported by the MRC (MR/P012272/1 and MC_UU_12024/1), the Rosetrees Trust, and the National Institute of Health Research Oxford Biomedical Research Centre.
