# Supplementary material for: Decoding voluntary movements and postural tremor based on thalamic LFPs as a basis for closed-loop stimulation for essential tremor
Source: Brain Stimul. 2019 Jul-Aug;12(4):858–67. doi: 10.1016/j.brs.2019.02.011 (PMC6600875; doi:10.1016/j.brs.2019.02.011)
Supplement: Multimedia component 1 [file mmc1.pdf]

Supplementary Methods:

Surgical procedure

For patients in Oxford, intended coordinates for the ViM thalamus were 12-14 mm lateral from the midline (x-axis), 3-5 mm posterior to the midcommissural point in the y-axis, and at the level of the line between the anterior and the posterior commissure in the z-axis. Individual adjustments were made according to pre-operative stereotactic T2-weighted MRI so that the electrodes were 2-4 mms inside the thalamus relative to the boarder. The electrodes were placed about 3mm beyond target so that the middle contacts straddled VIM. Patients were awake during the surgery. Intra-operative macrostimulation and clinical evaluation of the patient was also carried out.

For patients in Cologne, the ventral lateral posterior (VLp) nucleus of the thalamus was targeted on the basis of Schaltenbrand-Wahren atlas coordinates. Standard coordinates for targeting the lower border of the VLp were as follows: (1) 3-5.5 mm posterior to the midcommissural point in the y-axis, (2) at the level of the line between the anterior and the posterior commissure in the z-axis, and (3) 11.5–15.5 mm lateral to midline (x-axis).

Thus the intended target coordinates were only minimally different between the two centres.

Pre-defined motor tasks

Three patients (Ox1, 6 and 7) performed a ‘cued gripping force’ task, during which patients were asked to grip a dynamometer (hand dynamometer, Biometrics) so that a bar position indicating the measured force matched a cued position displayed on the monitor (Supplementary Fig. 1A). Each grip lasted 6 seconds with an inter-trial interval of 4-5 seconds (randomised) and there were 25-30 trials in each session. One patient (Ox2) performed a finger joystick task, during which the patient

was prompted to move a joystick so that the cursor (a red dot displayed on a monitor) corresponding to the joystick position would match a target position (a green dot). For each trial, the green target jumped from the centre to one of eight potential positions, and stayed at the target position for 1 second before returning to the centre of the screen (Supplementary Fig. 1B). There were 100 trials in each session, with each movement lasting 1-1.5 seconds and an inter-trial interval of 2 – 2.5 seconds. One patient (Ox3) performed a 'cued button pressing' task, during which they were asked to press the same key on a keyboard using the index finger once they saw a cue presented on a monitor. In this task, there were 100 trials in each session with each movement lasting around 0.5 seconds and an inter-trial interval of 2.5 – 3 seconds. In addition, two other patients performed self-paced continuous movements. One of them (Ox4) performed blocks of continuous finger tapping; and another (Ox5) performed blocks of continuous wrist movements (extension and flexion of the wrist). Each block of movement lasted for 20-30 seconds with 20-30 seconds between the movement blocks.

Recording

For post-operative recordings in the Oxford cohort, ViM thalamic local field potentials were recorded using a TMSi Porti amplifier (monopolar, common average reference, anti-aliasing low-pass filtering with a cut-off frequency of 500 Hz and sampling frequency of 2048Hz, TMS International, Netherlands) in patient Ox1, 2, 6 and 7. In patient Ox3-5, bipolar derivations from adjacent contact pairs were recorded through an Analog-to-digital-converter (1401power mk-II, Cambridge Electronic Design, Cambridge, UK) after amplification (Digitimer D360, Digitimer, Welfortshire, UK). Electromyography (EMG) was simultaneously recorded from the flexor and extensor carpi radialis in gripping movements, and from the first dorsal interosseous muscle (FDI) in finger joystick or finger tapping movements. Direct behavioural measurements such as generated gripping force (Biometrics hand dynamometer) or joystick positions were also simultaneously recorded using the same amplifier.

In addition, 3D accelerometers (± 3g, TMSi, Netherlands) were attached to the hand in order to measure kinematic movements of the hand and the presence of tremor.

For intraoperative recordings in Cologne, a commercially available recording system (Inomed Micro Electrode Recording System; software: MER 2.4 beta) was used. Two to five micro-macroelectrodes were used, selected from a central electrode and four concentrically configured (anterior, medial, posterior, and lateral) further electrodes with a distance of 2mm from the central electrode. Local field potentials from the macroelectrodes were recorded while the electrodes were in the surgical target. Activity of the extensor digitorum communis (EDC) and flexor digitorum longus (FDL) muscles of the contralateral forearm were also simultaneously recorded using surface EMG electrodes. Both LFP and EMG signals were bandpass filtered between 0.5 and 1 kHz during the recording and sampled at 2.5 KHz.

Labelling of movement states based on behavioural measurements

In the Oxford cohort, direct behavioural measurements (force or joystick position) or EMG were used to identify the period of time with or without movements. EMG activities were high-pass filtered with cut-off frequency of 1 Hz, rectified and smoothed within a moving time window of 0.2 s. All behavioural measurements were normalised: gripping force was normalised to the maximal gripping force measured before the task started; the joystick position was normalised to maximal possible displacement when the joystick was displaced to its extreme; EMG activities were normalised to the 95th percentile value of the recording session. The mean and standard deviation of the background behavioural measurements during a 10 s time window with the patient at rest were quantified. For each recording session, time points with behavioural measurements over Mean + 4*STD relative to the resting baseline were labelled as 'Movement'. Supplementary Fig. 2 shows examples of the recordings for different tasks with labelled 'movements' shown as thin black lines. All analyses were performed in MATLAB (v. 2016a, The MathWorks Inc., Natick, Massachusetts).

In the Cologne cohort, EMG activities were used to identify periods of time with or without postural tremor. EMG activities were high pass filtered with cut-off frequency of 1 Hz and rectified. Time-frequency decomposition of the rectified EMG activities was obtained by applying continuous Morlet wavelet transforms with a linear frequency scale ranging from 1 Hz to 46 Hz and constant number (= 6) of cycles across all calculated frequencies. The mean and standard deviation of the peak power in the 3-7 Hz frequency band in the EMG activity when each patient was at rest were quantified. Time points in the forearm elevated blocks with EMG 3-7 Hz activity over Mean + 4*STD were labelled as 'Postural tremor'.

Logistic Regression based Binary Classifier

Here we adopted the logistic regression (LR) based binary (two-class) classifier. The logistic regression model predicts the probability of the presence of movements or tremor at the current time point *t* (*p(t)*) based on the linear combination of a set of predictor variables:

$$log\left[\frac{p(t)}{1-p(t)}\right] = y(t) = \sum_{m=0}^{M}\sum_{k=1}^{K} w_{k,m} \cdot x_k(t-\tau_m) + w_0 \qquad (1)$$

Where *K* and *M* are the number of features and the number of time lags during which the features were taken into account for the prediction, respectively, $x_k(t-\tau_m)$ is the *k*th predictor variable with time lag τ_m relative to the current time point and $w_{k,m}$ is the associated weight, w_0 is an intercept constant representing the baseline probability of the occurrence of movements, and y(t) is the weighted sum of all different features. A monotonic, S-shaped continuous function (the Logistic function) was then used to map *y(t)* which ranges from -∞ to +∞ into a value between 0 and 1 ($p(t)$):

$$p(t) = \frac{1}{1+e^{-y(t)}} \qquad (2)$$

A threshold was then applied to *p(t)* to classify the current observation as movement/tremor or not.

In this study, the ROC was plotted and the AUC was quantified to evaluate classifier performance. The Receiver Operating Characteristics (ROC) curve plots the true positive rate (Sensitivity) against

false positive rate (1-Specificity) for different thresholds. The area under the ROC curve (AUC) provides a measure of the ability of the classifier to distinguish between the two states with 0.5 indicating a chance-level accuracy and 1 suggesting perfect classification.

Training and cross-validation of the Logistic Regression based Binary Classifier

Five-fold cross-validation was applied to each recording session. In each iteration, 4 folds (80% of data) were used to train the classifier, i.e. to determine the weight $w_{k,m}$ attributed to each predictor variable $x_k(t-\tau_m)$. All the weights form the weight vector ($\boldsymbol{w}$) with the length of K*M, with K and M the number of frequency bands and the number of time lags taken in to account in the logistic regression model, respectively. The weight vector ($\boldsymbol{w}$) was estimated using an optimisation function (*fminunc*) in Matlab to minimise a cost function ($J(\boldsymbol{w})$), which was related to mis-classification compared to the labelled state (L_t):

$$\boldsymbol{w} = \underset{\boldsymbol{w}}{\operatorname{argmin}} J(\boldsymbol{w})$$

$$J(\boldsymbol{w}) = -\frac{1}{N}\{\sum_{t=1}^{N}[L_t \cdot \log(p(t)) + (1-L_t) \cdot \log(1-p(t))]\}$$

Where N is the total time points of the training data, L_t is the labelled movement state of each time point according to behavioural measurements, and $p(t)$ is the output of the LR classifier of the time point given the weight vector w.

After the weight vector $\boldsymbol{w}$ was estimated based on the training data, the predictor variables extracted from the ViM thalamic LFPs recorded in the remaining 20% data were used to decode movement probability and to test the classifier performance. The training and testing were performed for five iterations so each data point is used for testing for once. The estimated weight vectors from the five iterations were averaged before evaluating the contributions of different LFP features in decoding.

For cross-task validation, the model with the averaged weight vector from the five iterations was applied to another independent dataset recorded while the patients performed voluntary movements different from those used for model training.

Supplementary Figure 1 and Legend

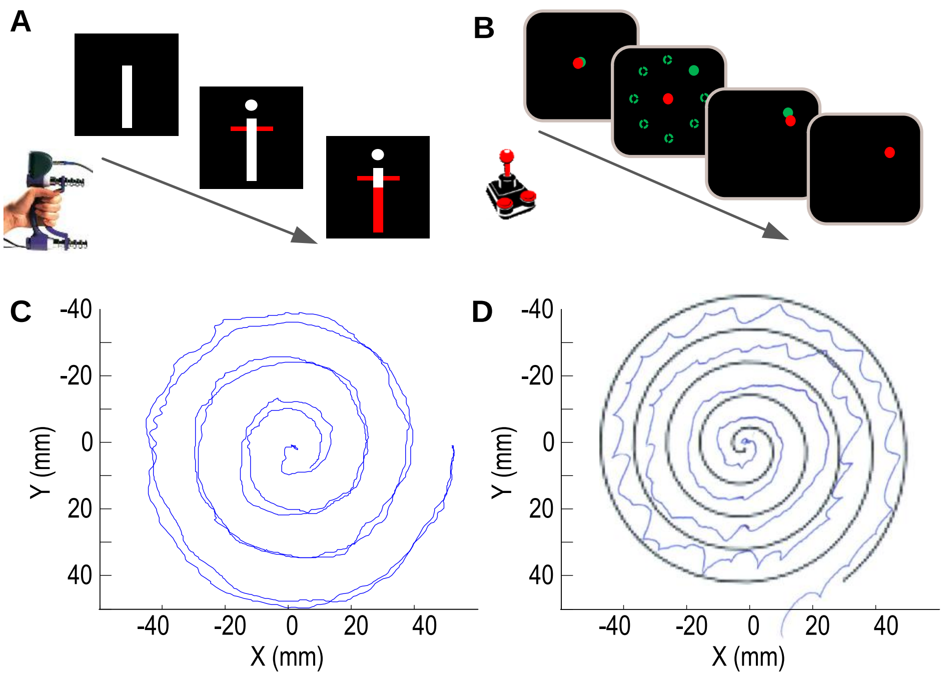

Supplementary Figure 1 Examples of voluntary movements tested in the study. A) Cued hand gripping movements in 3 patients; B) Finger joystick movements in 1 patient with both hands separately; C) and D) Self-paced spiral drawing used for cross-task validation in 4 patients. Spiral drawing also revealed variable degrees of tremor in the immediate post-operative period.

Supplementary Figure 2 and Legend

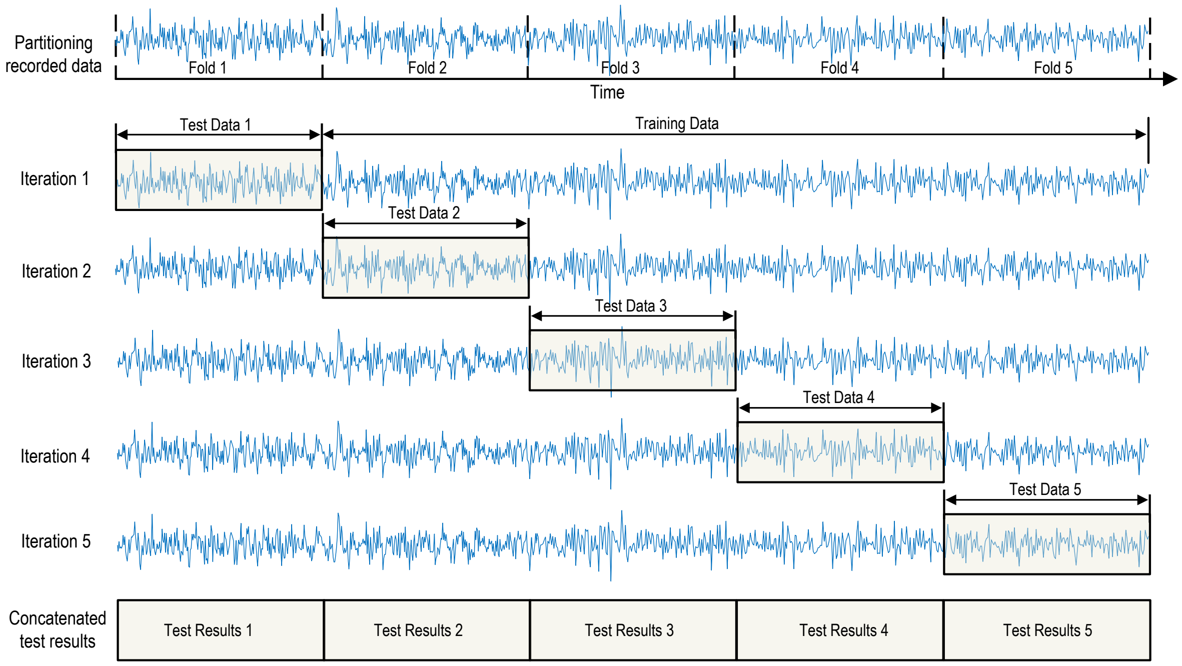

Supplementary Figure 2 Schematic of 5-fold cross-validation. Recorded data from a session (shown in blue line) was partitioned into 5 folds with each fold consisting of 20% of the continuous data. Five iterations of training and testing were performed. In each iteration, 4 folds (80% of data) were used to train the classifier. The performance of the classifier was tested on the remaining 20% data (in shaded area). With five iterations, each time point would be used for testing just once. The test results from the five iterations were concatenated to form the decoded movement probability for one complete recording session.

Supplementary Figure 3 and Legend

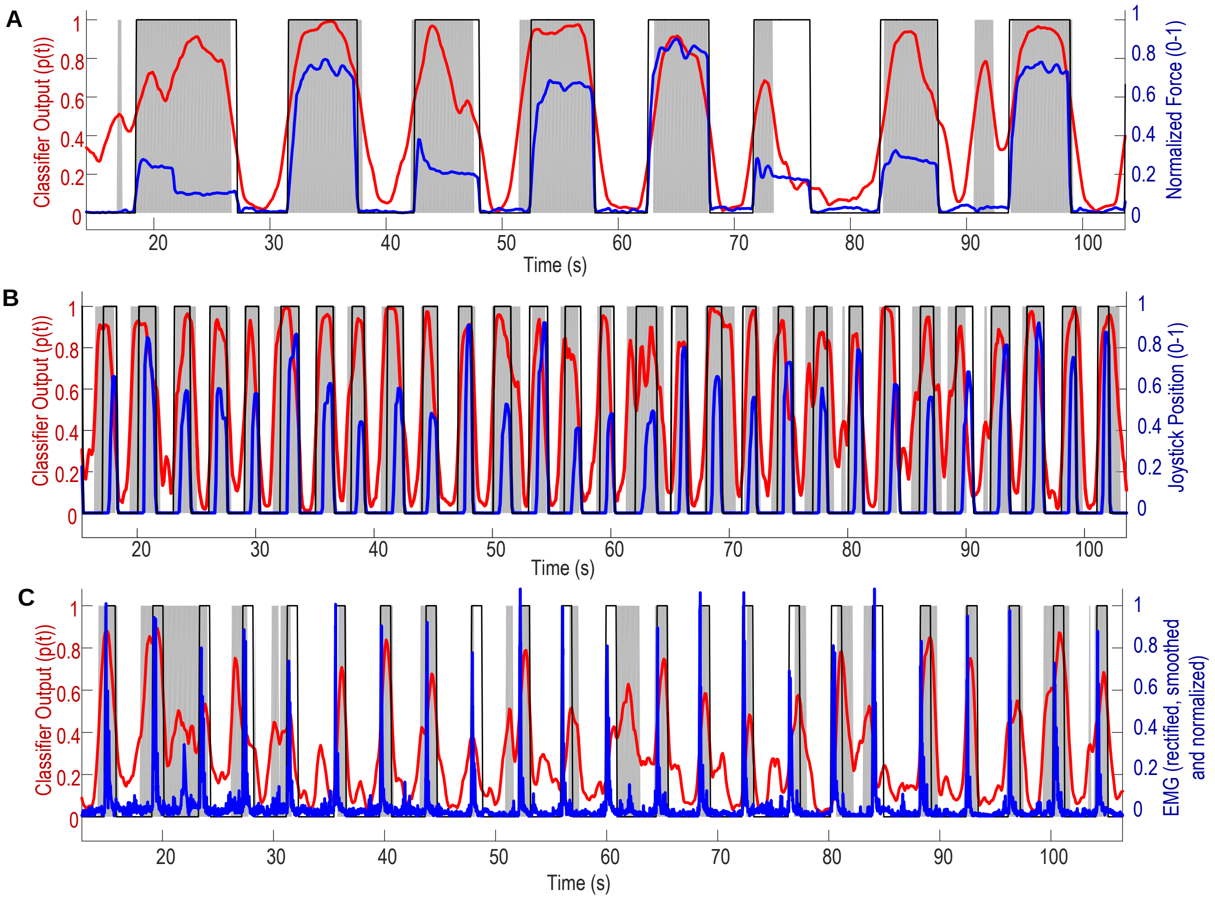

Supplementary Figure 3 Individual brief cued movements can be decoded based on ViM LFPs. A) Hand gripping from patient Ox1; B) Finger joystick movements from patient Ox2; C) Button pressing from patient Ox3. Blue lines are behavioural measurements. These are normalized gripping force in plot A, joystick displacement relative to the centre position in Plot B, and rectified and smoothed electromyography (EMG) in Plot C. Thin black lines show the time points with movements labelled according to behavioural measurements. Red lines are the classifier output (concatenated test results from the five iterations) indicating the decoded probability of movement. The grey shaded areas are the time points when the decoded probability was above 0.4. Normalisation was to the maximum value for force and displacement, and to the 95th percentile for EMG.

Supplementary Figure 4 and Legend

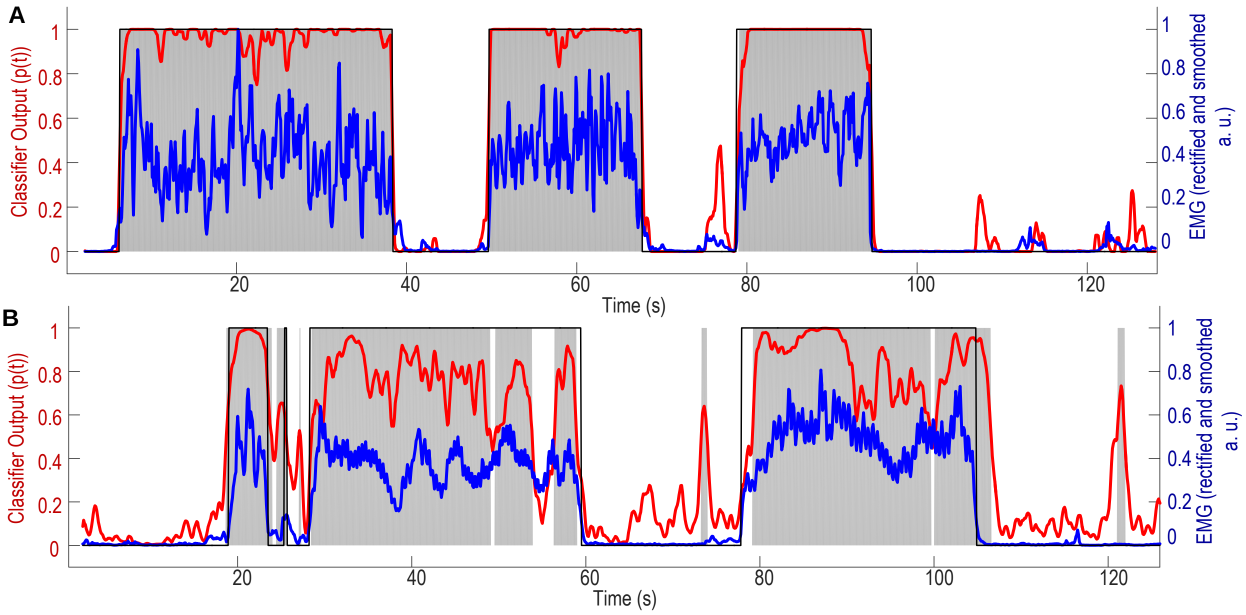

Supplementary Figure 4 Blocks of self-paced continuous movements can be decoded based on ViM LFPs. A) Continuous finger tapping was tested in patient Ox4; B) Extension and flexion of wrist was tested inpatient Ox5. Blue lines show normalised rectified and smoothed electromyography (EMG) activity. Thin black lines show the time points with movements labelled according to EMG. Red lines are the classifier output (concatenated test results from the five iterations) indicating the decoded probability of movement. The grey shaded areas are the time points when the decoded probability was above 0.4.

Supplementary Figure 5 and Legend

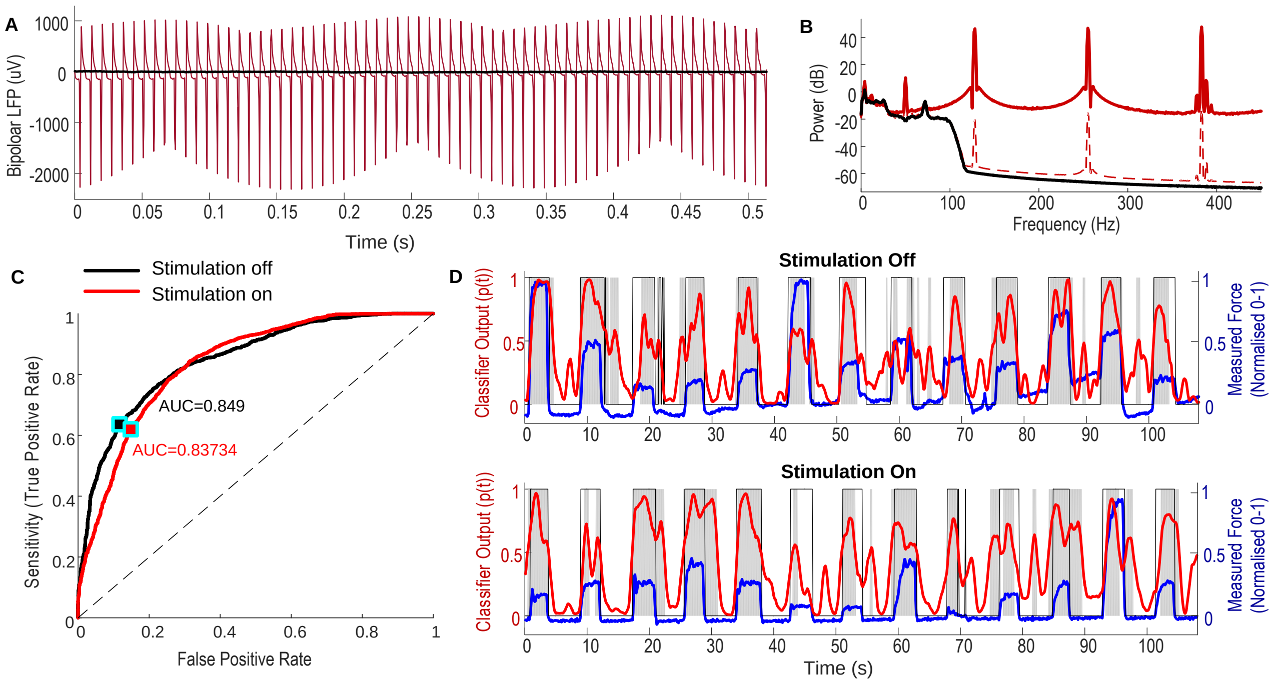

Supplementary Figure 5 Movements can be decoded when stimulation is switched on despite stimulation artefact. **A)** Monopolar high frequency stimulation (130 Hz) induced artefacts in the bipolar LFP measurements from the thalamus. Red and black lines show the bipolar LFP measurements with stimulation On and Off, respectively. Stimulation induced artefact can be a few thousand times the amplitude of the real signal. **B)** The power spectra of the thalamic LFPs when high frequency stimulation was switched on (red solid line) and off (black solid line). In this patient, the high frequency stimulation induced artefacts at all the harmonics of the stimulation frequency (130 Hz) in the frequency domain. A low pass filter (Chebyshev Type II IIR) with pass band up to 95 Hz and attenuation of 60 dB at 120 Hz reduced the power of the high frequency artefacts and was applied off stimulation (black solid line) and on stimulation (red interrupted line). **C)** ROC curves for movement decoding with stimulation ON (red line) and Off (black line). **D)** Individual movement was detected with similar accuracy for stimulation on (top panel) and simulation off (bottom panel). Blue lines show measured gripping force. Thin black lines show the time points with movements labelled according to measured force. Red lines are the classifier output (concatenated test results

from the five iterations) indicating the decoded probability of movement. The grey shaded areas are the time points when the decoded probability was above 0.4.
